# Competition and facilitation between the marine nitrogen-fixing cyanobacterium *Cyanothece* and its associated bacterial community

**DOI:** 10.3389/fmicb.2014.00795

**Published:** 2015-01-14

**Authors:** Verena S. Brauer, Maayke Stomp, Thierry Bouvier, Eric Fouilland, Christophe Leboulanger, Veronique Confurius-Guns, Franz J. Weissing, LucasJ. Stal, Jef Huisman

**Affiliations:** ^1^Department of Aquatic Microbiology, Institute for Biodiversity and Ecosystem Dynamics, University of AmsterdamAmsterdam, Netherlands; ^2^Department of Theoretical Biology, Center for Ecological and Evolutionary Studies, University of GroningenGroningen, Netherlands; ^3^Laboratoire Ecologie des Systèmes Marins Côtiers ECOSYM, UMR 5119, CNRS, IRD, Ifremer, Université Montpellier 2Montpellier, France; ^4^Department of Marine Microbiology, Royal Netherlands Institute for Sea ResearchYerseke, Netherlands

**Keywords:** aerobic anoxygenic phototrophs, cyanobacteria, heterotrophic bacteria, microbiota, nitrogen fixation, phytoplankton, resource competition, species interactions

## Abstract

N_2_-fixing cyanobacteria represent a major source of new nitrogen and carbon for marine microbial communities, but little is known about their ecological interactions with associated microbiota. In this study we investigated the interactions between the unicellular N_2_-fixing cyanobacterium *Cyanothece* sp. Miami BG043511 and its associated free-living chemotrophic bacteria at different concentrations of nitrate and dissolved organic carbon and different temperatures. High temperature strongly stimulated the growth of *Cyanothece*, but had less effect on the growth and community composition of the chemotrophic bacteria. Conversely, nitrate and carbon addition did not significantly increase the abundance of *Cyanothece*, but strongly affected the abundance and species composition of the associated chemotrophic bacteria. In nitrate-free medium the associated bacterial community was co-dominated by the putative diazotroph *Mesorhizobium* and the putative aerobic anoxygenic phototroph *Erythrobacter* and after addition of organic carbon also by the Flavobacterium *Muricauda*. Addition of nitrate shifted the composition toward co-dominance by *Erythrobacter* and the Gammaproteobacterium *Marinobacter*. Our results indicate that *Cyanothece* modified the species composition of its associated bacteria through a combination of competition and facilitation. Furthermore, within the bacterial community, niche differentiation appeared to play an important role, contributing to the coexistence of a variety of different functional groups. An important implication of these findings is that changes in nitrogen and carbon availability due to, e.g., eutrophication and climate change are likely to have a major impact on the species composition of the bacterial community associated with N_2_-fixing cyanobacteria.

## INTRODUCTION

Nitrogen is one of the key elements limiting the primary productivity of large parts of the oceans (Vitousek and Howarth, 1991; Moore et al., 2013). Hence, the input of new nitrogen by dinitrogen (N_2_)-fixing cyanobacteria will not only benefit the diazotrophs themselves but may also affect other members of the oceanic plankton community. For example, in a laboratory study, Agawin et al. (2007) demonstrated that the unicellular diazotroph *Cyanothece* sp. facilitated the non-diazotroph *Synechococcus* sp. through the release of fixed nitrogen. Furthermore, field studies have shown transfer of fixed nitrogen from large diazotrophic cyanobacteria to picoplankton in the Baltic Sea (Ohlendieck et al., 2000) and in the Southwest Pacific (Garcia et al., 2007). Similarly, blooms of diazotrophic *Trichodesmium* spp. are often succeeded by diatoms (Lenes et al., 2001) or dinoflagellates (Walsh and Steidinger, 2001; Mulholland et al., 2006), which may benefit from the nitrogen fixed by the diazotrophs.

It seems likely that marine N_2_-fixing cyanobacteria supply nitrogen not only to other phototrophs but also to chemotrophic bacteria. Yet, not much is known about the interactions between these two groups of organisms. Although *Trichodesmium* colonies are typically associated with chemotrophic bacteria (Paerl et al., 1989; Sheridan et al., 2002), both positive and negative interactions of N_2_-fixing cyanobacteria with chemotrophic bacteria have been described. For instance, Tseng et al. (2005) reported a higher abundance and productivity of chemotrophic bacteria during a *Trichodesmium* bloom. In contrast, Renaud et al. (2005) observed a decreased bacterial abundance and activity during a *Trichodesmium* bloom and also Nausch (1996) observed lower bacterial abundance and thymidine incorporation within a *Trichodesmium* bloom compared to the adjacent water.

The relationships between N_2_-fixing cyanobacteria and chemotrophic bacteria may indeed be complex. On the one hand, N_2_-fixing cyanobacteria and chemotrophic bacteria compete for dissolved nutrients such as phosphate. Chemotrophic bacteria are likely to be superior competitors for dissolved nutrients due to their small size and high substrate affinities (Currie and Kalff, 1984; Bratbak and Thingstad, 1985; Tambi et al., 2009). On the other hand, N_2_-fixing cyanobacteria can release substantial amounts of fixed nitrogen and organic carbon, which would facilitate chemotrophic bacteria (e.g., Glibert and Bronk, 1994; Mulholland et al., 2004, 2006; Wannicke et al., 2009; Dron et al., 2012; but see Benavides et al., 2013).

Furthermore, interactions within the microbial consortium are probably temperature dependent. N_2_-fixing cyanobacteria of the open ocean occur in significant numbers only in the warm waters of the tropics and subtropics (Stal, 2009). In an attempt to explain this (sub)tropical distribution we have demonstrated that in the unicellular N_2_-fixing *Cyanothece* sp. temperatures below 21°C hampered N_2_-fixation and induced nitrogen deficiency (Brauer et al., 2013). The cost per molecule of N_2_ fixed increased at low temperature, basically because the N_2_-fixation rate declined while the cells still needed to invest in an oxygen-free environment to enable functionality of the nitrogenase enzyme. Moreover, below 21°C the onset of nitrogenase activity was strongly delayed to the end of the night, probably because *de novo* synthesis of nitrogenase takes more time at low temperature. Higher temperatures decreased the cost of N_2_-fixation and stimulated growth and N_2_-fixation rates (Brauer et al., 2013). Hence, it is likely that increasing temperatures also increase the amount of nitrogen and organic carbon released by the diazotrophs, thereby stimulating chemotrophic bacteria indirectly.

With this study we aim for a better insight into the functioning of a microbial consortium consisting of an N_2_-fixing cyanobacterium and its associated chemotrophic bacteria. We formulate four testable hypotheses: (1) N_2_-fixing cyanobacteria are strongly stimulated by temperature, but will respond much less to an increase in dissolved nitrogen or organic carbon as they can fix N_2_ and CO_2_. (2) Chemotrophic bacteria associated with N_2_-fixing cyanobacteria are co-limited by dissolved nitrogen and organic carbon, and will strongly increase when dissolved nitrogen and organic carbon are either released by the diazotrophs or supplied in the growth medium. (3) Higher temperatures increase the abundance of chemotrophic bacteria because of enhanced nitrogen and organic carbon release by the N_2_-fixing cyanobacteria. (4) The taxonomic composition of the chemotrophic community changes with both temperature and nutrient availability.

We tested these hypotheses by growing the marine unicellular N_2_-fixing cyanobacterium *Cyanothece* sp. together with its accompanying community of chemotrophic bacteria in chemostats under different nitrogen, dissolved organic carbon and temperature conditions.

## MATERIALS AND METHODS

### *CYANOTHECE* STRAIN

The marine unicellular cyanobacterium *Cyanothece* sp. strain Miami BG043511 (further named *Cyanothece*) is a facultative diazotroph that assimilates nitrate and switches to N_2_-fixation when nitrate becomes scarce (Agawin et al., 2007). The non-axenic but unialgal strain was obtained from the University of Hawaii Culture Collection and maintained at room temperature under moderate light conditions in carbon and nitrogen-deficient mineral medium (“-C-N”; see below).

### CHEMOSTAT EXPERIMENTS

*Cyanothece* was grown in chemostats together with its associated community of chemotrophic bacteria. Chemostats were made of glass tubes with a water jacket and had an inner diameter of 4 cm and a working volume of approximately 250 mL. The water jacket was connected to a refrigerating-heating circulator (F12-EH, Julabo GmbH, Seelbach, Germany), which guaranteed precise temperature control (±0.1°C) of the culture. Light was supplied from one side at an alternating 12:12 h light:dark cycle and an incident light intensity of 110 μmol photons m^-2^ s^-1^, which is a saturating light level for the growth of *Cyanothece* (Brauer et al., 2013). Chemostats were run at a dilution rate of 0.2 d^-1^ using a peristaltic pump (Minipuls 2, Gilson, Inc., Middleton, WI, USA). Bubbling of the culture vessels with air from a six-channel aquarium pump (ACO-9620, Guangdong Hailea Group Co., Ltd, Raoping, China) assured homogeneous mixing of the culture.

*Cyanothece* and its accompanying chemotrophic bacterial community were cultured at three temperatures (18, 23, and 28°C) and four different nutrient regimes. The nutrient regimes were determined by the nitrogen and dissolved organic carbon (DOC) content of the growth medium, which contained either 0 or 100 μmol L^-1^ NaNO_3_ and either 0 or 2 mmol L^-1^ carbon provided as glucose. The medium always contained 25 μmol L^-1^ KH_2_PO_4_. The concentrations of the nutrients were chosen such that DOC, nitrate, or phosphate may become growth limiting at steady-state. All other nutrients were available in excess (Supplementary Table 1). Hence, the molar C:N:P ratios in the four different nutrient treatments were 0:0:1 (“-C-N”), 0:4:1 (“-C+N”), 80:0:1 (“+C-N”), and 80:4:1 (“+C+N”). Sampling of the chemostats started the day after inoculation.

Unfortunately, one treatment (28°C; – C+N) failed. *Cyanothece* cell density and the steady state measurements of particulate organic phosphorus (POP) were recovered from a previous pilot experiment performed under the same conditions but other data are missing.

### CELL COUNTS AND BIOVOLUME

Samples (1.75 mL) for cell counts were taken almost every day and fixed with formaldehyde (1% final concentration) for 30 min, flash-frozen with liquid nitrogen, and stored at -80°C. Cells were stained with SYBR-Green 1 (Lumiprobe, FL, USA) and counted in triplicate by flow cytometry (FACS Calibur equipped with a 488 nm laser, Becton Dickinson, San Jose, CA, USA) using pre-calibrated counting beads (2.07 μm Nile Red, Spherotech, Inc., Lake Forest, IL, USA). The flow cytometer discriminated between cyanobacteria and other bacteria based on fluorescence and light scatter. Cell numbers of *Cyanothece* and chemotrophic bacteria are reported as the mean of the triplicate counts. Steady state cell numbers were calculated as the mean over the last 10 days of each experiment (day 22–32).

The total biovolume of *Cyanothece* and chemotrophic bacteria at steady state was calculated as the product of steady state cell number and the mean cell volume. Because volume data of individual cells were right-skewed, mean cell volume was estimated as the geometric mean of 30 (*Cyanothece*) or 100 (chemotrophic bacteria) cells. Individual cell volume was calculated from the measurements of length *L* and width *W* of SYBR-Green 1-stained cells with epifluorescence microscopy. Cell volume *V*_C_ of a prolate spheroid *Cyanothece* cell was calculated as *V*_C_ = (π/6) ×*W*^2^ × L (Hillebrand et al., 1999). Cell volume *V*_b_ of a coccus or rod-shaped bacterium was calculated as *V*_b_ = (π/4) ×*W*^2^ × (*L*-*W*/3) (Bratbak, 1985).

### NUTRIENT DYNAMICS

For the determination of residual DOC (glucose), N (nitrate), and P (phosphate) concentrations 2 mL (for DOC) or 6 mL (for N and P) of culture were sampled almost every day and filtered over a sterile 0.2 μm membrane filter (Acrodisc; Pall Corporation, Port Washington, NY, USA; for C) or a glass fiber filter (GF/F, Whatman, Maidstone, UK; for N and P). The filtrates were stored at -20°C. Nitrate and phosphate concentrations were analyzed by an autoanalyzer (SEAL QuAAtro, SEAL Analytical, ltd., Hampshire, UK). DOC (glucose) concentrations were determined with the glucose-oxidase assay (GAGO-20, Sigma–Aldrich, USA). Steady state nutrient concentrations were calculated as mean concentration between day 22 and day 32 of each experiment.

### ELEMENTAL COMPOSITION OF THE COMMUNITY

The elemental composition of the total community was determined from the concentrations of particulate organic carbon (POC), particulate organic nitrogen (PON), and POP at steady state. Measurements were done in triplicate by filtering 5 mL of culture over a pre-combusted glass fiber filter (Whatman GF/F; for POC and PON) or over a 0.45 μm nitrocellulose membrane filter (Millipore Corporation, Bedford, MA, USA; for POP), previously washed with 0.2 M HCl and rinsed with demineralized water. Filters were subsequently rinsed again with demineralized water and stored at -20°C. Prior to analysis, filters were dried at 60°C for 48 h. POC and PON were analyzed by an element analyzer (NA-2500 Thermo Analyzer, Thermo Fisher Scientific, Whaltham, MA, USA). POP was determined through digestion of the organic material with nitric acid at 200°C and subsequent analysis at 213.6 nm with an inductively coupled plasma-optical emission spectrophotometer (iCap 6000 ICP-OES Analyzer, Thermo Fisher Scientific).

### CHEMOTROPHIC BACTERIA CLONE LIBRARY

The composition of the chemotrophic bacterial community at steady state was determined with the help of 16S rRNA gene clone libraries. A sample of 60–90 mL was taken from each chemostat and filtered over a glass fiber filter (Whatman GF/C; nominal pore size 1.2 μm) to remove *Cyanothece* cells. The smaller bacteria that passed through were collected on a 0.2 μm polycarbonate filter (Nuclepore Track-Etched Membrane, Whatman) and stored at -20°C. Filters were cut into small pieces and bacterial DNA was extracted using the MOBIO UltraClean Soil DNA extraction kit (MOBIO Laboratories, Inc. Carlsbad, CA, USA) according to the manufacturer’s protocol. The concentration of the isolated DNA was measured spectrophotometrically with a NanoDrop ND 1000 (Nanodrop Technologies, Inc. Wilmington, DE, USA). The nearly complete 16S rRNA gene was then amplified using the primers 8F (5′ AGA GTT TGA TCM TGG CTC AG 3′) and 1492R (5′ GGT TAC CTT GTT ACG ACT T 3′) (Weisburg et al., 1991). The 25 μl PCR reaction mixture contained 3% v/v dimethyl sulfoxide (Sigma-Aldrich, Munich, Germany), 0.01% v/v bovine serum albumin (Fermentas, Hanover, MD, USA), 0.2 μmol of each primer, 0.2 μmol dNTP’s (Roche Applied Science, Indianapolis, IN, USA), 1x HotStarPCR buffer and 0.04 units HotStarTaq DNA Polymerase (Qiagen Inc., Valencia, CA, USA), and 10–20 ng DNA. Cycling conditions were 15 min at 95°C, followed by 35 cycles with 1 min at 95°C, 30 s at 55°C, and 1 min and 50 s at 72°C, and a final extension period of 7 min at 72°C. PCR products were separated by electrophoresis on a 1% w/v agarose (Sigma-Aldrich) gel and stained with SYBR Gold (Invitrogen Corp., Carlsbad, CA, USA). Amplicon size was estimated by comparison with a Mass Ruler DNA Ladder (Fermentas) and purified using EZNA Cycle Pure Kit (Omega bio-tek Inc., Doraville GA, USA). The freshly purified amplicons were cloned with the help of the TOPO-TA cloning Kit (Invitrogen Corp.). White colonies were selected, suspended in 10 μL MilliQ water, and boiled for 10 min to free the vectors. The inserted 16S rRNA gene fragment was amplified with the vector primers M13F (5′ GTA AAA CGA CGG CCA G 3′) and M13R (5′ CAG GAA ACA GCT ATG AC 3′). The 25 μL PCR mixture contained 0.2 μmol of each primer, 0.2 μmol dNTP’s, 1x PCR reaction buffer and 0.03 units NEB Taq polymerase (New England Biolabs Inc., Ipswich, MA, USA), and 1 μL of sample DNA. Cycling conditions were 2 min at 94°C, followed by 40 cycles with 1 min at 94°C, 1 min at 55°C, and 2 min at 72°C, and a final extension period of 10 min at 72°C. The PCR products were checked on a 1% agarose gel. Amplicons were purified using Sephadex G-50 Superfine (Sigma-Aldrich) and DNA-concentrations were determined spectrophotometrically by NanoDrop. Amplicons were partially sequenced (400–600 bp) using the 1492R-primer and the BigDye Terminator chemistry (Big Dye Terminator v3.1 Cycle Sequencing Kit, Applied Biosystem, Foster City, CA, USA) according to the manufacturer’s protocol, using a 3130 Genetic Analyzer (Applied Biosystems).

### NUCLEOTIDE SEQUENCE ACCESSION NUMBERS

The newly determined 16S rRNA sequences were deposited in NCBI GenBank under accession numbers KP325715 – KP326298.

### STATISTICS AND SEQUENCE ANALYSIS

To test the effects of temperature and nutrient treatment, a two-way ANOVA without replication (McDonald, 2009; Zar, 2010) was conducted on the mean of cell numbers and biovolumes measured between day 22 and 32 of the experiment (steady-state). A normal two-way ANOVA was applied to the independent replicates of POC, PON, and POP. Because of the failed treatment (28°C; –C+N) all ANOVAs were calculated using Type IV sum of squares, which are designed for the situation where there are missing cells. ANOVAs were performed in SPSS 22.0.0.

Sequences were manually inspected and corrected in MEGA 6.06 and analyzed for similarity in BLASTn (Basic Local Alignment Search Tool, National Center for Biotechnology Information, 8600 Rockville Pike, Bethesda, USA). To compare the community composition of associated chemotrophic bacteria between the treatments, a matrix of pairwise Euclidean distances was calculated based on shifted log-transformed (1+x) absolute taxon abundance. The distance matrix was subjected to a hierarchical agglomerative cluster analysis using the complete linkage algorithm. The cluster analysis was done in R 3.0.3.

## RESULTS

### POPULATION DYNAMICS

**FIGURE 1:**
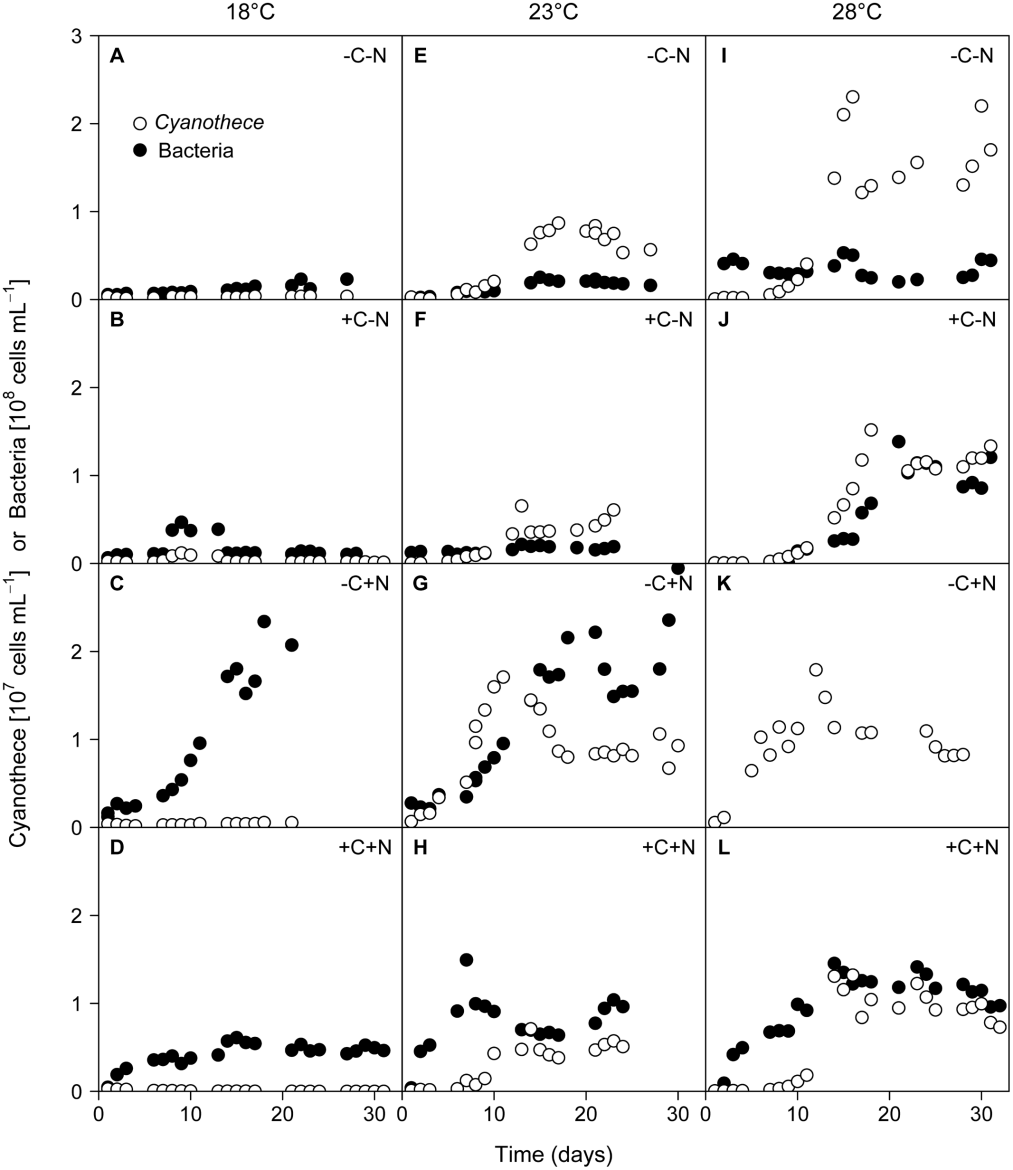
**Time series of *Cyanothece* sp. and chemotrophic bacteria.** *Cyanothece* sp. (open symbols) and chemotrophic bacteria (closed symbols) were grown together in chemostat experiments. Columns show temperature treatments at **(A–D)** 18°C, **(E–H)** 23°C, and **(I–L)** 28°C, rows show nutrient treatments with or without addition of DOC (C) and nitrate (N). Data of chemotrophic bacteria from the failed -C+N treatment at 28°C (panel K) are missing.

Regular microscopic inspection revealed that the microbial consortium in our experiments consisted of healthy non-senescent *Cyanothece* cells and free-living chemotrophic bacteria; we did not observe bacteria attached to *Cyanothece* cells or other forms of cell aggregates. The growth of *Cyanothece* was hardly affected by the nutrient treatment, but was strongly affected by temperature (**Figure 1**). At 18°C, the population density of *Cyanothece* remained at a low but constant level in three of the four treatments (–C–N, +C–N, –C+N; **Figures 1A–C**), whereas it was continuously declining in the other treatment (+C+N; **Figure 1D**). At 23 and 28°C, the population density of *Cyanothece* initially increased and then approached a steady state after 10 to 18 days in all nutrient treatments (**Figures 1E–L**).

Conversely, chemotrophic bacteria showed a weaker response to temperature but a strong response to the nutrient treatment, in particular to nitrate addition. When neither DOC nor nitrate was added to the medium (–C–N) chemotrophic bacteria density remained low throughout the experiment at all temperatures (**Figures 1A,E,I**). Addition of DOC (+C–N) did not lead to an increase of chemotrophic bacteria at the lower temperatures (**Figures 1B,F**) but stimulated bacteria at 28°C (**Figure 1J**). In response to nitrate addition (–C+N) bacteria exhibited strong growth and reached high population densities after 16–18 days at both 18 and 23°C (**Figures 1C,G**). Bacterial data are not available for the –C+N treatment at 28°C. The simultaneous supply of DOC and nitrate (+C+N) generally stimulated bacteria, with higher population densities at a higher temperature (**Figures 1D,H,L**). Bacterial densities in the +C+N treatment were generally lower than in the –C+N treatment (compare **Figures 1D,H,L** with **Figures 1C,G,K**).

**FIGURE 2:**
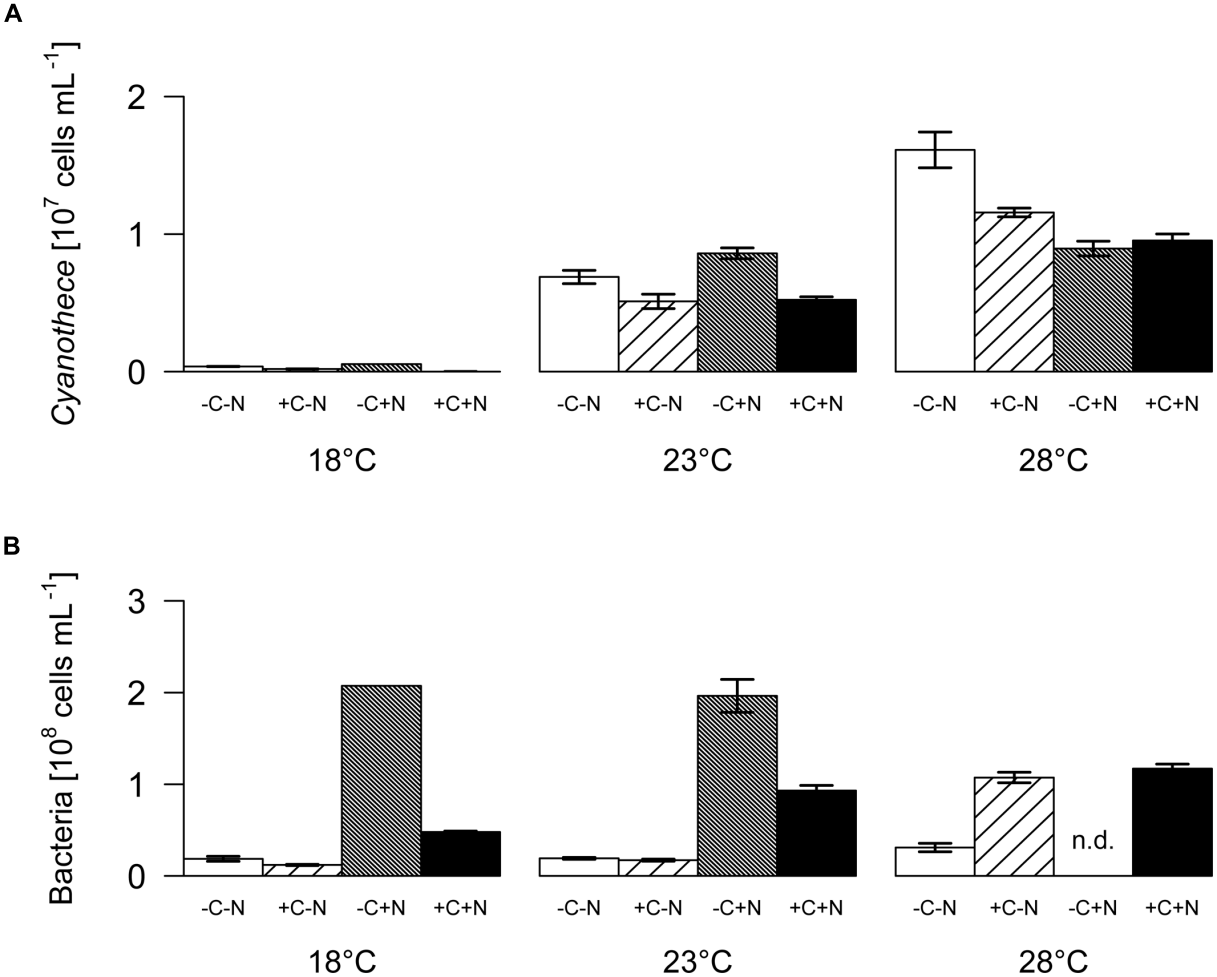
**Steady-state population densities. (A)** *Cyanothece* sp., **(B)** chemotrophic bacteria. Bars show the mean ± SE of the population densities measured between day 22 and day 32, for each temperature and nutrient treatment (see **Figure 1**). Nutrient treatments: +C = with added DOC; +N = with added nitrate. SE are not available for the -C+N treatment at 18°C (*n* = 1); n.d. = no data.

The steady state population density of *Cyanothece* was significantly influenced by temperature but not by nutrient treatment (**Figure 2A**; temperature: *F*_2,6_ = 28.778, *p* < 0.001; nutrient treatment: *F*_3,6_ = 1.017, *p* = 0.448). Conversely, the steady state population density of chemotrophic bacteria was significantly affected by nutrient treatment but not by temperature (**Figure 2B**; temperature: *F*_2,6_ = 4.297, *p* = 0.082; nutrient treatment: *F*_3,6_ = 26.015, *p* = 0.002). The steady state biovolume of *Cyanothece* and bacteria showed the same patterns as the steady state population density (Supplementary Figure 1).

**FIGURE 3:**
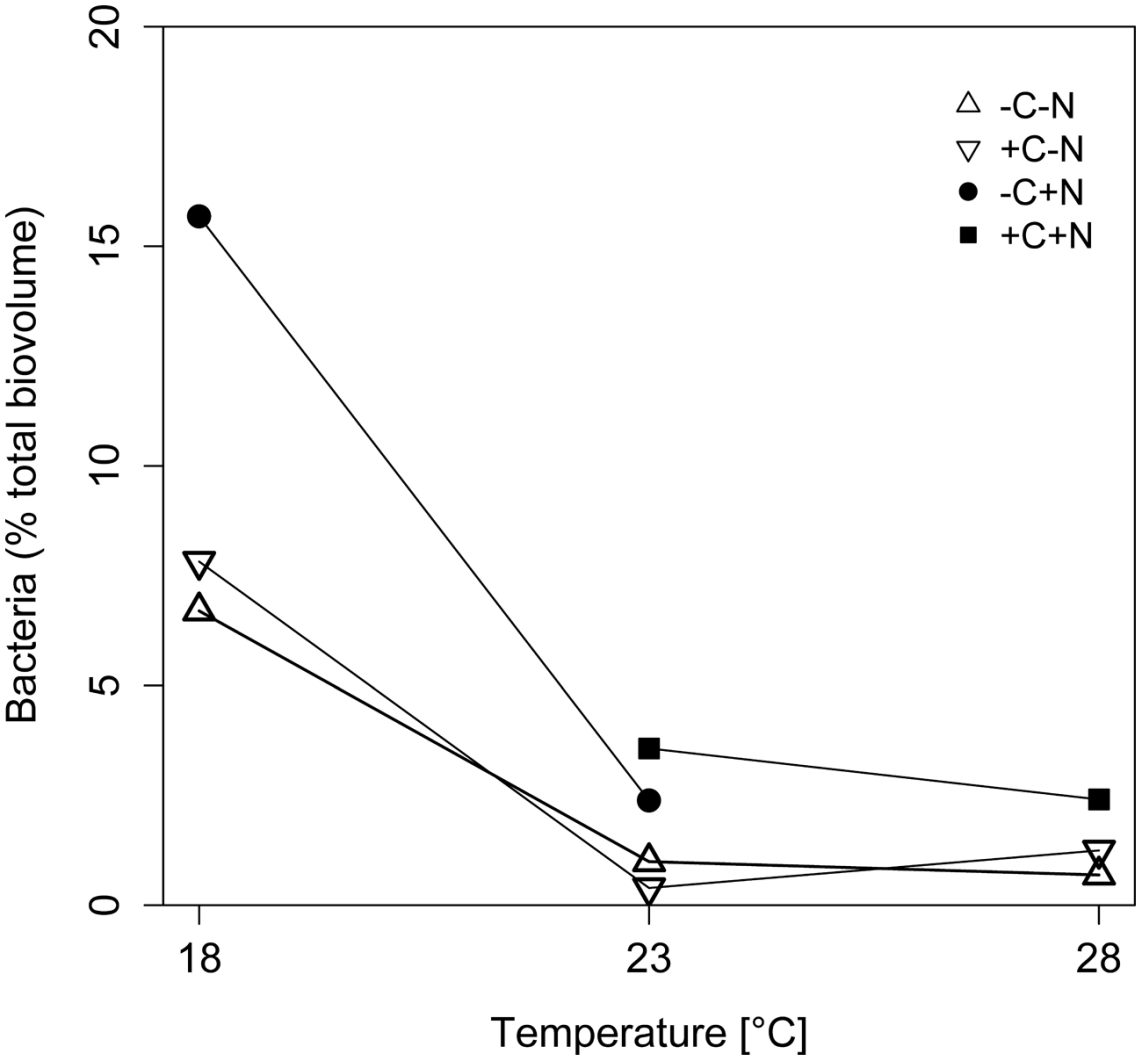
**Effect of temperature on the relative abundance of chemotrophic bacteria.** The relative abundance of chemotrophic bacteria is expressed as percentage of the total biovolume at steady state. Symbols indicate different nutrient treatments (+C = with added DOC; +N = with added nitrate). Data points are missing for the +C+N treatment at 18°C because the *Cyanothece* abundance was too low to be accurately quantified, and for the failed -C+N treatment at 28°C.

The contribution of chemotrophic bacteria to the total community was not affected by the nutrient treatment but was significantly reduced at higher temperatures (**Figure 3**; temperature: *F*_2,6_ = 10.675, *p* = 0.025; nutrient treatment: *F*_3,6_ = 2.36, *p* = 0.213), from 7 to 18.5% of the total community biovolume at 18°C to < 4% at 23 and 28°C. Hence, in terms of biomass, at higher temperatures the community was almost completely dominated by *Cyanothece*.

### NUTRIENT DYNAMICS

**FIGURE 4:**
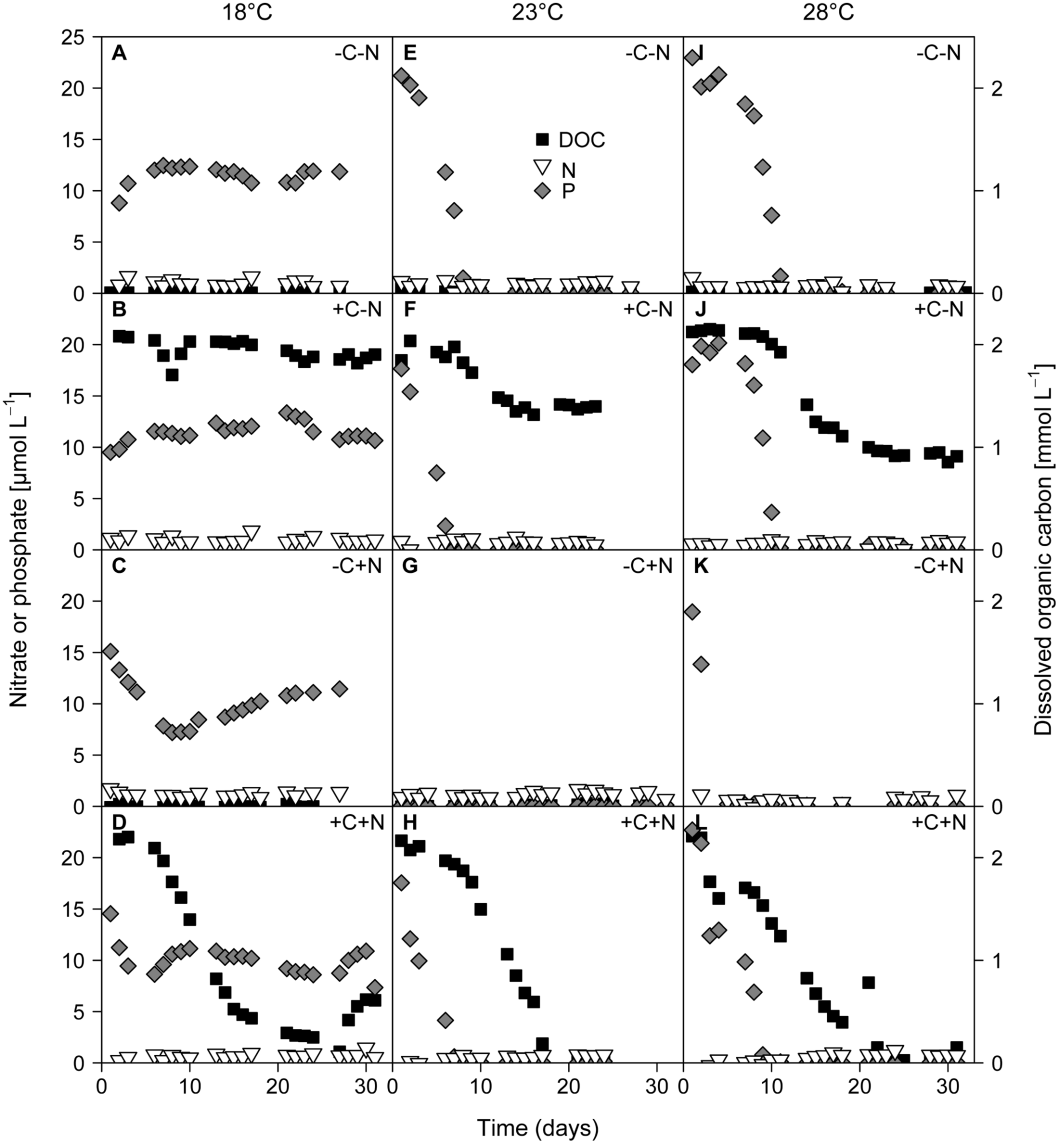
**Time series of residual concentrations of DOC (black squares), nitrate (white inverted triangles), and phosphate (gray diamonds) during the experiments.** Columns show temperature treatments at **(A–D)** 18°C, **(E–H)** 23°C and **(I–L)** 28°C, rows show nutrient treatments with or without addition of DOC (C) and nitrate (N). DOC data from the failed -C+N treatment at 28°C (panel K) are missing.

The growth of *Cyanothece* and chemotrophic bacteria led to the depletion of DOC, nitrate, and phosphate (**Figure 4**). Nitrate concentrations were diminished to very low levels from the first day of sampling onward in all treatments. At 18°C phosphate concentrations remained high at ∼10 μmol L^-1^ (**Figures 4A–D**). In contrast, at 23 and 28°C phosphate was depleted to < 0.2 μmol L^-1^ within 1 to 10 days (**Figures 4E–L**). In the +C–N treatment the DOC concentration remained high at 18°C, and was only mildly depleted to 1.4 mmol C L^-1^ at 23°C and to 0.9 mmol C L^-1^ at 28°C (**Figures 4B,F,J**). In contrast, in the +C+N treatment the DOC concentration was strongly depleted to 0.4 mmol C L^-1^ at 18°C and to < 0.15 mmol C L^-1^ at 23 and 28°C (**Figures 4D,H,L**). The nutrient concentrations at steady state are summarized in Supplementary Figure 2.

### ELEMENTAL COMPOSITION OF THE COMMUNITY

**FIGURE 5:**
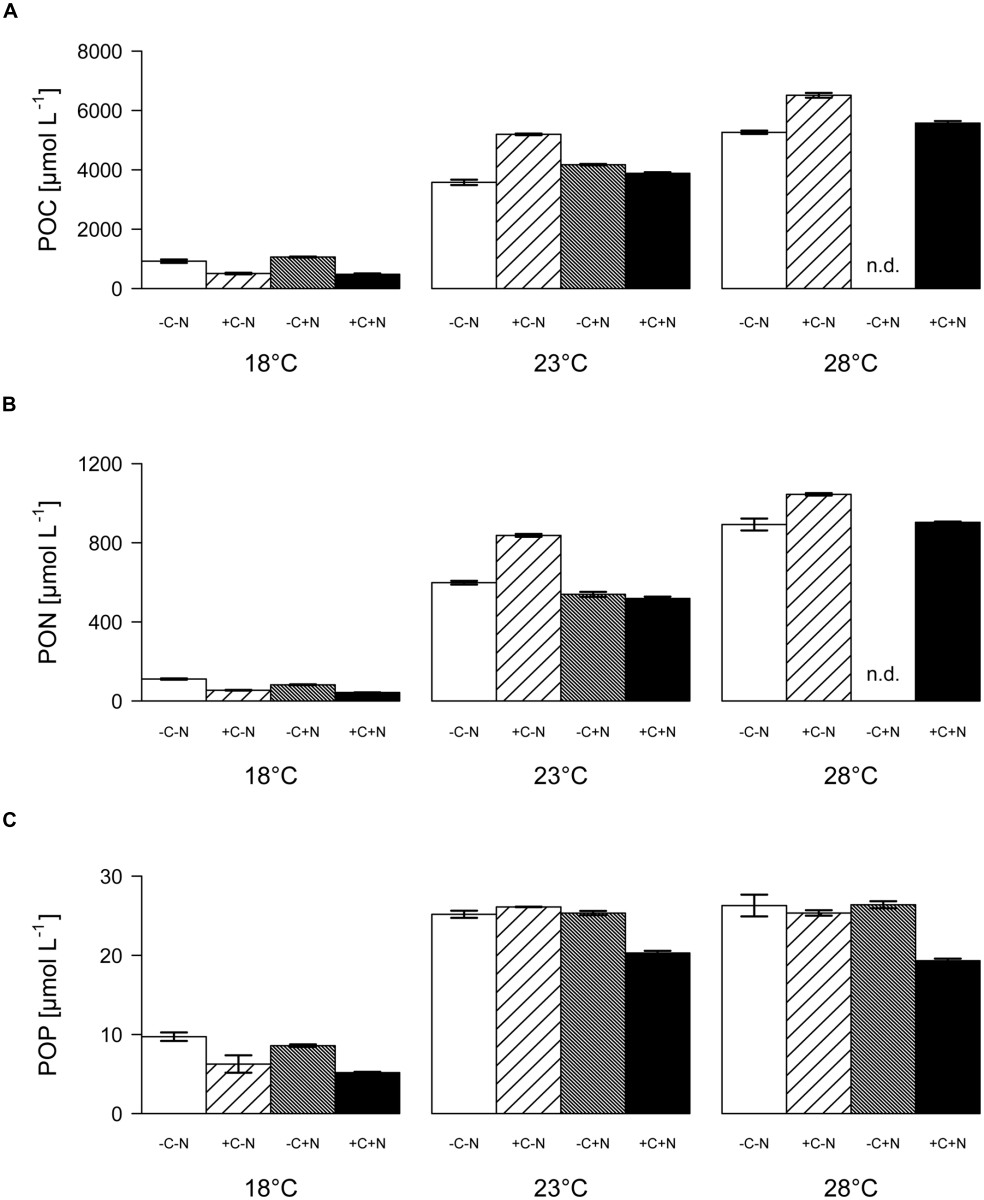
**Elemental composition of the community. (A)** POC, **(B)** PON, and **(C)** POP concentrations at steady state, for each temperature and nutrient treatment. Bars show means ± SE of triplicate measurements. Nutrient treatments: +C = with added DOC; +N = with added nitrate; n.d. = no data.

Both the POC and PON concentrations at steady state increased significantly with temperature but were not affected by nutrient treatment (**Figure 5A**; temperature: *F*_2,6_ = 90.77, *p* < 0.001; nutrient treatment: *F*_3,6_ = 1.691, *p* = 0.283; **Figure 5B**; temperature: *F*_2,6_ = 83.5, *p* < 0.001; nutrient treatment: *F*_3,6_ = 1.853, *p* = 0.255). POP concentrations were significantly affected by both temperature and nutrient treatment (**Figure 5C**; temperature: *F*_2,6_ = 218.025, *p* < 0.001; nutrient treatment: *F*_3,6_ = 11.151, *p* = 0.007). POP concentrations at 23 and 28°C reached values close to the phosphate concentration of 25 μmol L^-1^ of the supplied growth medium. Hence, almost all available phosphate had been taken up by the organisms, consistent with the depleted phosphate concentrations in the cultures at 23 and 28°C (**Figures 4E–L**). POP concentrations were slightly but significantly lower in the +C+N treatment than in the other treatments (*post hoc* comparison of the means with Tukey HSD: *p* < 0.01 for +C+N vs. -C-N; *p* < 0.05 for +C+N vs. +C-N and for +C+N vs. -C+N).

**FIGURE 6:**
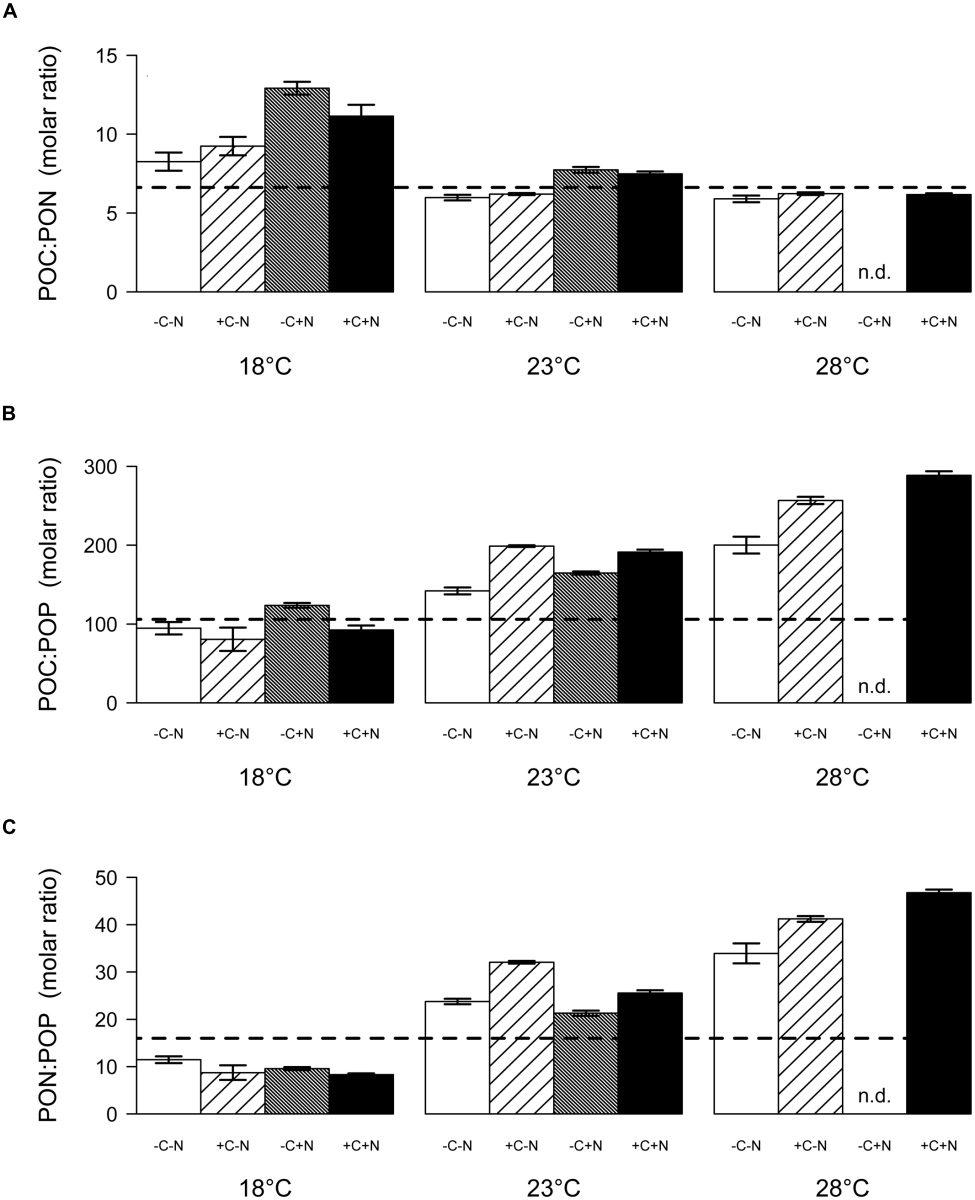
**Steady-state elemental ratios (in mol/mol) of the entire community. (A)** POC:PON ratio, **(B)** POC:POP ratio, and **(C)** PON:POP ratio. Bars show the mean ± SE of triplicate measurements, for each temperature and nutrient treatment. Horizontal dashed lines indicate the Redfield ratio of C:N:P = 106:16:1. Nutrient treatments: +C = with added DOC; +N = with added nitrate; n.d. = no data.

The molar ratio of POC:PON at steady state decreased significantly with temperature but was only marginally affected by nutrient treatment (**Figure 6A**; temperature: *F*_2,6_ = 22.016, *p* = 0.003; nutrient treatment: *F*_1,6_ = 5.18, *p* = 0.054). Conversely, the POC:POP and PON:POP ratios both increased significantly with temperature, and were not affected by the nutrient treatment (**Figure 6B**, temperature: *F*_2,6_ = 26.649, *p* = 0.002; nutrient treatment: *F*_1,6_ = 1.606, *p* = 0.3; **Figure 6C**, temperature: *F*_2,6_ = 35.548, *p* = 0.001; nutrient treatment: *F*_1,6_ = 0.711, *p* = 0.586). As a consequence, the C:N:P stoichiometry of the community was invariant with respect to nutrient treatment but changed with temperature from 100:10:1 at 18°C, to 174:26:1 at 23°C, to 238:39:1 at 28°C.

### COMMUNITY COMPOSITION OF CHEMOTROPHIC BACTERIA

**FIGURE 7:**
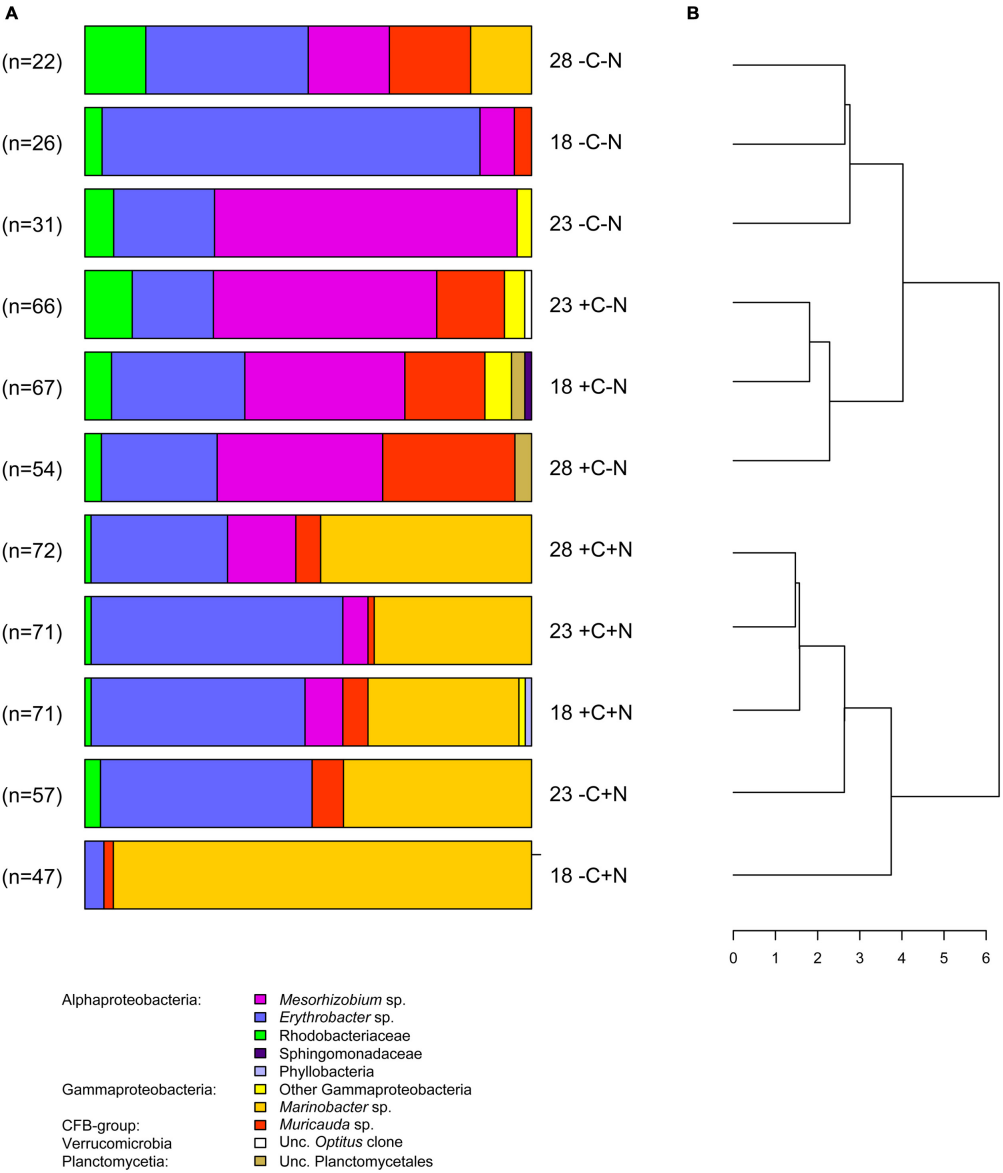
**Community composition. (A)** Community composition of the chemotrophic bacteria at steady state, for each temperature and nutrient treatment. Different colors indicate different taxonomic groups. Treatments were with or without addition of DOC (C) and nitrate (N) at three different temperatures (18, 23, and 28°C). Data from the failed -C+N treatment at 28°C are missing. **(B)** Dendrogram derived from cluster analysis of the community composition.

In total, 584 partial sequences of the bacterial 16S rRNA gene were analyzed by BLASTn, revealing 21 different OTUs. A separate PCR of the archaeal 16S rRNA gene yielded insignificant amounts of amplified DNA, indicating that archaea were not important in our experiments. Of the bacteria, 567 sequences revealed similarities between 97 and 100% with known organisms, while the other 17 sequences exhibited similarities between 93 and 96%. When possible, OTUs were grouped at the genus level, except the genera *Roseobacter*, *Labrenzia,* and one unknown *Rhodobacteriaceum*, which were grouped as Rhodobacteriaceae. Furthermore, the genera *Alcanivorax*, *Halomonas,* and one unknown Gammaproteobacterium were assigned as “other Gammaproteobacteria.” The number of sequences analyzed per treatment varied between 22 and 72, which yielded 3–7 different taxonomic groups per treatment (**Figure 7A**). Each chemotrophic community was co-dominated by 2–4 genera from three major taxonomic groups, i.e., the Alphaproteobacteria *Erythrobacter* and *Mesorhizobium*, the Gammaproteobacterium *Marinobacter*, and the Flavobacterium *Muricauda*. An additional clone library detected *nifH* genes belonging to the order Rhizobiales, which indicates that *Mesorhizobium* possessed the genetic equipment for N_2_-fixation.

The bacterial community composition at steady state varied strongly with nutrient treatment but was less affected by temperature (**Figure 7A**). In the treatments without nitrate (-C-N, +C-N), *Erythrobacter* and *Mesorhizobium* were co-dominant and the communities also contained a smaller but consistent proportion of Rhodobacteriaceae. Addition of DOC to the nitrate-free treatments increased the relative abundance of *Muricauda*. *Erythrobacter* and *Marinobacter* co-dominated the communities in treatments with nitrate (-C+N, +C+N). There was a small but consistent increase in the proportion of *Mesorhizobium* in the +C+N treatments compared to the -C+N treatments. Cluster analysis grouped the bacterial communities according to nitrogen treatment at the highest level of the dendrogram and then further subdivided these communities according to the carbon and temperature treatment (**Figure 7B**).

## DISCUSSION

In this study we investigated the interactions between a small unicellular N_2_-fixing cyanobacterium and its associated community of chemotrophic bacteria. All bacteria were free living according to our observations based on light and fluorescence microscopy, although this should be interpreted with some caution because higher-resolution methods such as scanning electron microscopy may have revealed bacteria attached to the *Cyanothece* cells. Nevertheless, it appears that there was little cell-to-cell contact that may have facilitated direct transfer of carbon and nutrients between the unicellular cyanobacteria and the other community members. In this sense, our study differs from earlier work on the bacterial communities of much larger filamentous diazotrophic cyanobacteria such as *Trichodesmium*, *Nodularia* and *Aphanizomenon*, where the bacteria were often attached to the filaments (Paerl, 1984; Paerl et al., 1989; Salomon et al., 2003; Tuomainen et al., 2006; Ploug et al., 2010, 2011; Hmelo et al., 2012). We will now discuss our findings in relation to the four hypotheses that were formulated in the introduction.

### HYPOTHESIS 1: TEMPERATURE AND NUTRIENT EFFECTS ON *CYANOTHECE*

The chemostat experiments confirm our first hypothesis that the steady state abundance and biomass of *Cyanothece* depends strongly on temperature, and less on nutrient treatment (**Figure 2A**). Moreover, *Cyanothece* largely dominated the microbial community in terms of biomass (**Figure 3**). Hence, several community-wide parameters such as particulate carbon, nitrogen and phosphorus, as well as their elemental ratios were mainly driven by *Cyanothece* and therefore responded strongly to temperature but not to nutrient treatment (**Figures 5** and **6**).

Interestingly, *Cyanothece* appeared to be limited by phosphorus at higher temperatures but by nitrogen at lower temperatures. At 18°C phosphate was not depleted, the PON:POP ratio dropped below the Redfield ratio of 16 and the POC:PON ratio rose beyond the Redfield ratio of 6.6. Hence, at low temperature N_2_-fixation did not meet the nitrogen demand of *Cyanothece*. This conclusion is consistent with previous results, which showed that temperatures below 21°C hampered N_2_-fixation and provoked a strong increase in the cellular C:N ratio of *Cyanothece* (Brauer et al., 2013). Addition of nitrate at 18°C did not alleviate nitrogen limitation of *Cyanothece* because nitrate was primarily taken up by chemotrophic bacteria. The increase in *Cyanothece* abundance with temperature coincided with an increase in the PON:POP ratio beyond the Redfield ratio and a drop in the POC:PON ratio below Redfield, which suggest that N_2_-fixation met and even outreached the nitrogen demand (**Figures 2** and **6**). At 23 and 28°C virtually all phosphate was depleted and incorporated into particulate matter (**Figures 4** and **5**). Thus, increasing temperature enhanced the N_2_-fixation activity of *Cyanothece*, which alleviated *Cyanothece* from nitrogen limitation and caused a switch to phosphate limitation.

The temperature dependence of *Cyanothece* observed in our experiments is in agreement with the global distribution of marine photoautotrophic diazotrophs, which are largely confined to (sub)tropical oceans (Capone et al., 1997; Staal et al., 2003; Stal, 2009). Previously, we demonstrated that this global distribution might be caused by strong increases in the physiological cost of nitrogen fixation at temperatures below 21°C (Brauer et al., 2013). While this former study was conducted under nutrient-saturated conditions, we here show that the temperature dependence of diazotrophs also persists at low phosphate levels. Since iron and phosphate often limit N_2_-fixation in the open ocean (Sañudo-Wilhelmy et al., 2001; Mills et al., 2004), our results provide additional support for the hypothesis that the global distribution of marine N_2_-fixing cyanobacteria in the pelagic is limited by temperature (Staal et al., 2003; Stal, 2009).

### HYPOTHESIS 2 AND 3: TEMPERATURE AND NUTRIENT EFFECTS ON CHEMOTROPHIC BACTERIA

The strong growth response to nitrate addition and the weak response to DOC addition contradicted our second hypothesis that chemotrophic bacteria should be co-limited to a similar extent by both nitrogen and carbon. The bulk of chemotrophic bacteria were clearly limited by nitrogen. However, even though the DOC concentration was markedly depleted in the +C+N treatment (**Figures 4D,H,L**), comparison with the -C+N treatment indicates that DOC addition had a negative impact on bacterial growth (**Figure 2B**). Possibly the high DOC concentration had inhibitory effects on the growth of the chemotrophic bacteria.

The presence of chemotrophic bacteria in treatments without added nitrate or DOC indicates that *Cyanothece* facilitated its accompanying bacterial community through the release of dissolved nitrogen and carbon. Yet, it seems that *Cyanothece* released relatively more carbon than nitrogen in comparison to the C:N requirements of chemotrophic bacteria, because the bulk of chemotrophic bacteria were primarily limited by nitrogen. The C:N ratio of marine bacteria varies approximately from 4:1 under carbon limitation to 13:1 under nitrogen limitation (Goldman and Dennett, 2000). Little is known about the C:N release ratio of diazotrophs. However, it is noteworthy that *Cyanothece* sp. CCY 0110 has one of the highest production rates of extracellular polymeric substances measured so far (Mota et al., 2013), and our personal observations revealed that *Cyanothece* sp. Miami BG043511 has a tendency of high mucus production. This hints at a particularly high carbon release rate in the genus *Cyanothece*, which would be consistent with our hypothesis that *Cyanothece* released relatively more organic carbon than nitrogen.

The nitrogen release rates by *Cyanothece* also seemed to be low in absolute terms. Assuming a nitrogen content of 5 fg N cell^-1^ (Allen et al., 2005) chemotrophic bacteria in the nitrate-deficient cultures incorporated 0.7–8% of the total particulate nitrogen, with an average of 3.4%. Similarly, low nitrogen release rates have recently been inferred from the quantification of external nitrogen pools in cultures of *Cyanothece* sp. Miami BG043511 (Benavides et al., 2013). Yet, these low values disagree with many other studies demonstrating very high nitrogen release rates by the filamentous *Trichodesmium*, ranging between 12 and 82% of recently fixed nitrogen (Mulholland, 2007). It is also in contrast with the study of Agawin et al. (2007) who found that *Cyanothece* sp. Miami BG043511 promoted a fourfold increase of the non-diazotrophic *Synechococcus* sp. through nitrogen release. One explanation for these contrasting findings might be that nitrogen release rates are generally less high in unicellular diazotrophs than in the filamentous *Trichodesmium* (Benavides et al., 2013). Furthermore, in our study *Cyanothece* was limited by phosphorus, while in the study of Agawin et al. (2007) it was limited by light, which means that its physiological state differed between the two experiments. In *Trichodesmium* low phosphorus concentrations have been shown to decrease specific N_2_-fixation rates (Fu and Bell, 2003; Mulholland and Bernhardt, 2005; but see Wannicke et al., 2009). It is conceivable that in our experiments phosphorus limitation decreased the cellular N_2_-fixation rate of *Cyanothece* as well, resulting in a relatively low nitrogen release rate.

Our third hypothesis, which states that higher temperature increases the amounts of released nitrogen and organic carbon and thus the number of chemotrophic bacteria, was supported only to a certain extent. One treatment (-C+N) did not show an increase of the chemotrophic bacteria from 18 to 23°C, while we lack data at 28°C. Hence, it is difficult to draw any final conclusions on the temperature response of the chemotrophic bacteria for this treatment. In the other three nutrient treatments, the chemotrophic bacteria increased with temperature as predicted by our hypothesis. However, they increased less strongly with temperature than *Cyanothece*. Hence, at higher temperatures, chemotrophic bacteria made up only a small fraction of the entire community (**Figure 3**), indicating that facilitation by *Cyanothece* did not significantly increase with temperature.

In our experiments resource competition between *Cyanothece* and chemotrophic bacteria played a minor role in the treatments without added nitrogen, since *Cyanothece* was primarily limited by phosphate, while chemotrophic bacteria were limited particularly by combined nitrogen and partly by organic carbon. Hence, both groups of organisms occupied rather distinct niches. However, in the +N treatments chemotrophic bacteria were partially alleviated from nitrogen limitation and may have shifted to P limitation, which will have brought them into more direct competition with the P-limited *Cyanothece* population. This is visible in the decline of *Cyanothece* and increase in chemotrophic bacteria from day 10 to day 18 in the –C+N treatment (**Figure 1G**) and also in the lower steady-state abundance of *Cyanothece* in the +N treatments than in the -N treatments at 28°C (**Figure 2A**).

### HYPOTHESIS 4: CHANGES IN COMMUNITY COMPOSITION

The free-living chemotrophic bacteria in our experiments were dominated by *Erythrobacter*, *Mesorhizobium*, *Marinobacter,* and *Muricauda*, which are common inhabitants of the marine pelagic and of phytoplankton cultures (Schäfer et al., 2002; Kazamia et al., 2012; Le Chevanton et al., 2013). This suggests that the bacterial taxa were probably not specific for *Cyanothece*. This would be in line with Rooney-Varga et al. (2005), who showed that free-living bacteria found in association with phytoplankton are not very specific to these organisms. In contrast, bacteria attached to phytoplankton cells are often highly species-specific and may have evolved a long-term relationship with their hosts (Grossart et al., 2005; Rooney-Varga et al., 2005; Sison-Mangus et al., 2014).

We do not know whether our consortium represents a natural association of N_2_-fixing cyanobacteria and chemotrophic bacteria, or whether it is a laboratory artifact. However, a stable culture of a non-axenic cyanobacterium represents an excellent model system to study the interactions in this consortium. Moreover, most of the bacterial genera that we detected in our study are common in oceanic waters, and this gives confidence that the results obtained in this experimental study can be extrapolated to the natural environment.

Our results provide only partial support for Hypothesis 4. The taxonomic composition of the microbial community responded strongly to the nutrient treatments, in particular to nitrate addition, but was less affected by temperature. *Marinobacter* exhibited the clearest signs of nitrate limitation, as it was mostly absent in the nitrate-deficient cultures but reached high numbers in the nitrate-amended cultures (**Figure 7**). This is consistent with other studies describing *Marinobacter* as common nitrate-assimilating bacteria in marine habitats (Allen et al., 2005; Cai and Jiao, 2008). *Marinobacter* was not affected by DOC or temperature.

*Erythrobacter* was generally the most successful species and the only one that was able to persist in all experimental treatments. This fits the observation that members of this genus are ubiquitous in the photic zone of the ocean (Hügler and Sievert, 2011). *Erythrobacter* seemed to be slightly stimulated by nitrate but not by DOC addition. However, it was also abundant in the nitrate-deficient cultures, which means that it was also able to utilize other, *Cyanothece*-derived nitrogen compounds. *Erythrobacter* spp. might be good competitors for DOC compounds as many of them belong to the aerobic anoxygenic phototrophs (AAPs), which are photoheterotrophs containing bacteriochlorophyll *a* (Kolber et al., 2001; Koblížek et al., 2003; but see Oh et al., 2009). It has been shown that *Erythrobacter* sp. NAP1 uses light to increase its carbon use efficiency by substituting respiratory ATP-production with photophosphorylation (Hauruseu and Koblížek, 2012). Although similar observations have been made for the AAP-bacterium *Roseobacter* sp. COL2P belonging to the *Rhodobacteriaceae* (Hauruseu and Koblížek, 2012), the *Rhodobacteriaceae* in our study were much less successful than *Erythrobacter* (**Figure 7**).

*Mesorhizobium* sp. was mainly present in the nitrate-free cultures, although small numbers of *Mesorhizobium* were still present when both nitrate and DOC were supplied (**Figure 7**). The dominance of *Mesorhizobium* in nitrate-deficient environments and the presence of *nifH* genes belonging to the order Rhizobiales suggest that *Mesorhizobium* fixed dinitrogen. Hence, it was probably not limited by dissolved nitrogen but by organic carbon (energy-limited). This may also explain the presence of *Mesorhizobium* in the +C+N treatment. Although nitrate was added to the mineral medium, the nitrate concentration at steady state was depleted in the +C+N treatment (**Figures 4D,H,L**), thus favoring suitable growth conditions for diazotrophic growth of *Mesorhizobium* when provided with sufficient organic carbon.

*Muricauda* was present in almost all cultures but only reached significant numbers in nitrate-deficient cultures when DOC was added (**Figure 7**). This could hint at carbon- (energy) limitation. The abundance of *Muricauda* was stimulated by higher temperature, which agrees with its preference for aquatic environments between 25 and 30°C (Bruns et al., 2001; Hwang et al., 2009). As *Muricauda* belongs to the Flavobacteria, it was possibly degrading the high-molecular mass fraction of the *Cyanothece*-derived organic matter (see, e.g., Teeling et al., 2012).

Thus, a closer look at the response of the individual species reveals that not all chemotrophic bacteria were nitrogen limited, but that different species were controlled by different limiting resources. These interspecific differences indicate that differential resource utilization played an important role in this community and contributed to the coexistence of chemotrophic bacteria from a variety of functional groups. This supports the view that niche differentiation is a major determinant of bacterial diversity in the relatively homogeneous habitat of the open oceans (Hutchinson, 1961; Stomp et al., 2004; Hunt et al., 2008; Teeling et al., 2012). Hence, the bacterial species composition was neither neutral (*sensu* Hubbell, 2001) nor completely dictated by the diazotroph, but was structured by environmental conditions and species interactions in a similar way as many other ecological communities (Tilman, 1982; Prosser et al., 2007; Fuhrman, 2009; Brauer et al., 2012). An important implication is that changes in nitrogen and carbon availability due to, e.g., eutrophication and climate change are likely to induce shifts in the bacterial communities associated with N_2_-fixing cyanobacteria.

## Conflict of Interest Statement

The authors declare that the research was conducted in the absence of any commercial or financial relationships that could be construed as a potential conflict of interest.
