# Supplementary material for: Competition and facilitation between the marine nitrogen-fixing cyanobacterium Cyanothece and its associated bacterial community
Source: Front Microbiol. 2015 Jan 14;5:795. doi: 10.3389/fmicb.2014.00795 (PMC4294207; doi:10.3389/fmicb.2014.00795)
Supplement: Supplementary file 1 [file Image_1.PDF]

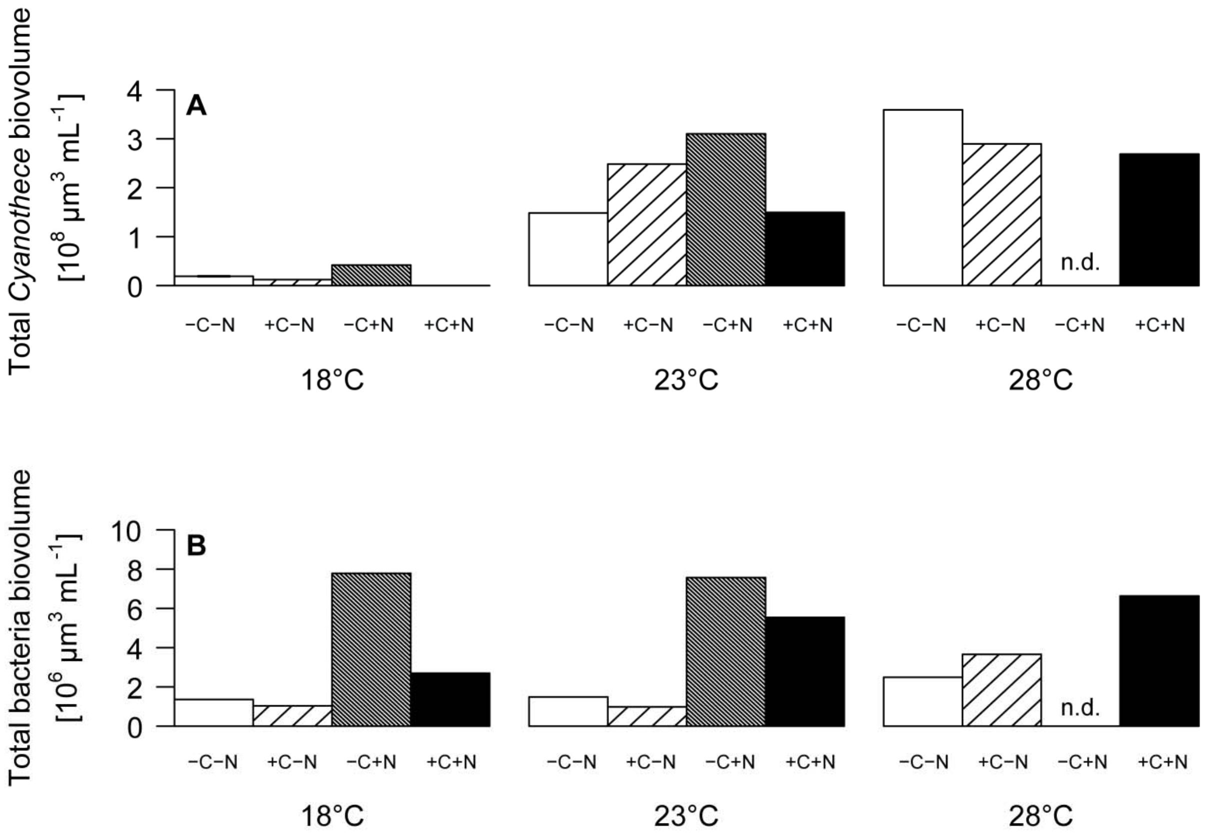

Supplementary Figure 1- Total biovolume of the community at steady-state. (A) *Cyanothece*, **(B)** chemotrophic bacteria. Bars show the geometric mean of the total biovolume measured between day 22 and day 32 of the experiment, for each temperature and nutrient treatment. Nutrient treatments: +C = with added DOC; +N = with added nitrate; n.d. = no data.
