# Supplementary material for: Competition and facilitation between the marine nitrogen-fixing cyanobacterium Cyanothece and its associated bacterial community
Source: Front Microbiol. 2015 Jan 14;5:795. doi: 10.3389/fmicb.2014.00795 (PMC4294207; doi:10.3389/fmicb.2014.00795)
Supplement: Supplementary file 2 [file Image_2.PDF]

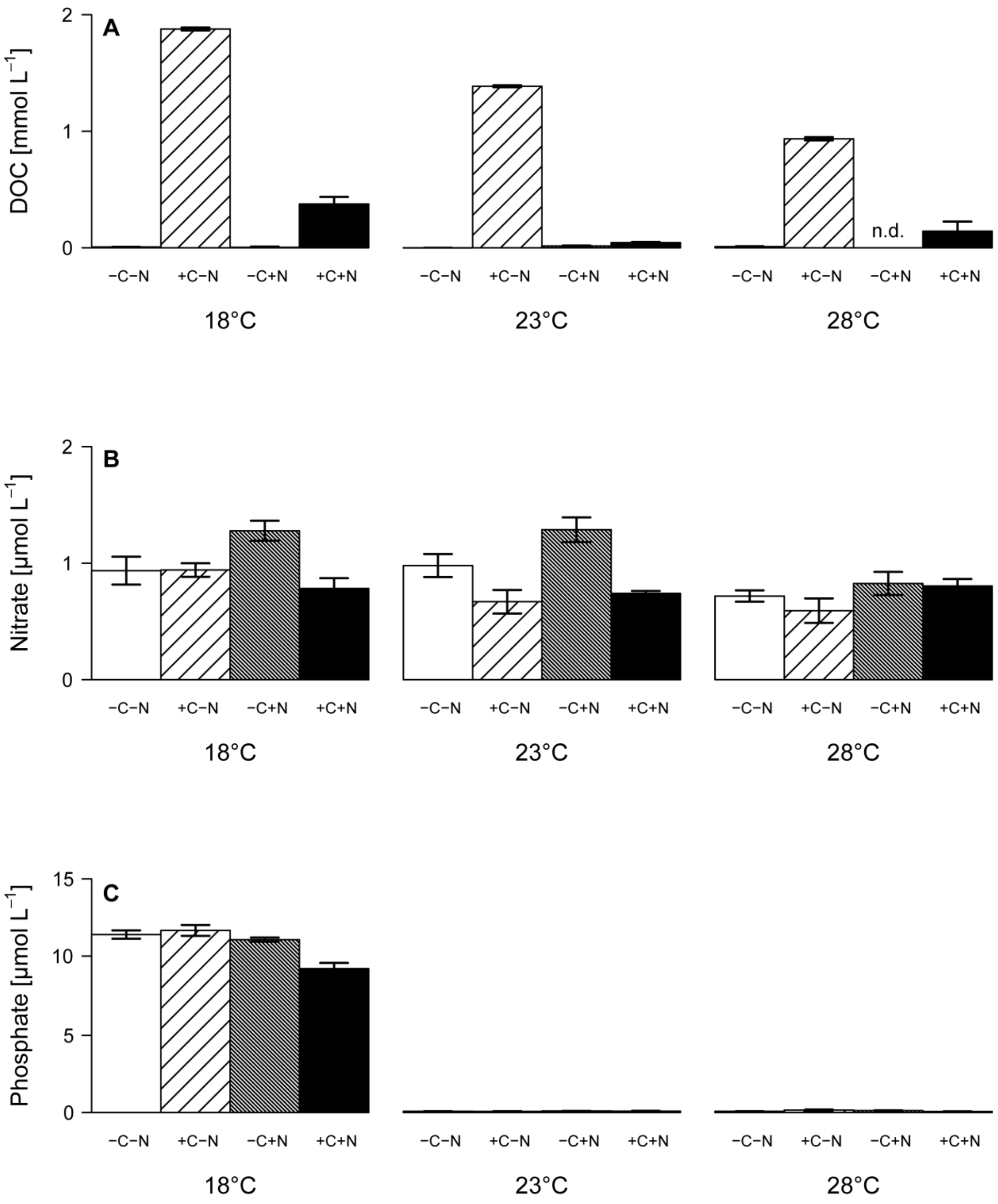

Supplementary Figure 2- Steady-state concentrations of the three major nutrients. (A) DOC, **(B)** nitrate, **(C)** phosphate. Bars show the mean ± SE of the nutrient concentrations measured between day 22 and day 32 of the experiment, for each temperature and nutrient treatment. Nutrient treatments: +C = with added DOC; +N = with added nitrate; n.d. = no data.
