# Supplementary material for: Competition and facilitation between the marine nitrogen-fixing cyanobacterium Cyanothece and its associated bacterial community
Source: Front Microbiol. 2015 Jan 14;5:795. doi: 10.3389/fmicb.2014.00795 (PMC4294207; doi:10.3389/fmicb.2014.00795)
Supplement: Supplementary file 3 [file Table_1.PDF]

## Supplementary Table 1 - Composition of the growth medium

| Compound | Concentration (µmol $L^{-1}$) |
|---|---|
| **Salts/Buffers:** | |
| $MgSO_4$•$7H_20$ | $2.0 \times 10^4$ |
| KCl | $8.0 \times 10^3$ |
| $CaCl_2$•$2H_2O$ | $2.5 \times 10^3$ |
| NaCl | $4.3 \times 10^5$ |
| $NaHCO_3$ | $2.0 \times 10^3$ |
| **Macro nutrients:** | |
| $C_6H_{12}O_6$ | 333; 0 |
| $NaNO_3$ | 100; 0 |
| $K_2HPO_4$•$3H_2O$ | 12.5 |
| $Na_2SiO_3$•$9H_2O$ | 160 |
| $H_3BO_3$ | 550 |
| **Micro nutrients:** | |
| $FeSO_4$•$7H_20$ | 14 |
| $Na_2EDTA$ | 35 |
| $MnCl_2$•$4H_2O$ | 22 |
| $ZnCl_2$ | 2.4 |
| $Na_2MoO_4$•$2H_2O$ | 5.4 |
| $CuSO_4$•$5H_2O$ | 0.2 |
| $CoCl_2$•$4H_2O$ | 0.5 |
| **Vitamins:** | |
| Thiamine•HCl (B1) | 0.6 |
| Biotin | $4.0 \times 10^{-3}$ |
| Cyanocobalamin (B12) | $7.4 \times 10^{-3}$ |
